# Digital droplet PCR and IDAA for the detection of CRISPR indel edits in the malaria species *Anopheles stephensi*

**DOI:** 10.2144/btn-2019-0103

**Published:** 2020-02-10

**Authors:** Rebeca Carballar-Lejarazú, Adam Kelsey, Thai Binh Pham, Eric P Bennett, Anthony A James

**Affiliations:** 1 Department of Microbiology & Molecular Genetics, University of California, Irvine, CA 92697-4025, USA; 2 Department of Odontology, Copenhagen Center for Glycomics, Faculty of Health Sciences, University of Copenhagen, Copenhagen DK-2200, Denmark; 3 Department of Molecular Biology & Biochemistry, University of California, Irvine, CA 92697-3900, USA

**Keywords:** CRISPR-Cas9, ddPCR, gene editing, IDAA, mosquitoes, NHEJ quantification

## Abstract

CRISPR/Cas9 technology is a powerful tool for the design of gene-drive systems to control and/or modify mosquito vector populations; however, CRISPR/Cas9-mediated nonhomologous end joining mutations can have an important impact on generating alleles resistant to the drive and thus on drive efficiency. We demonstrate and compare the insertions or deletions (indels) detection capabilities of two techniques in the malaria vector mosquito *Anopheles stephensi*: Indel Detection by Amplicon Analysis (IDAA™) and Droplet Digital™ PCR (ddPCR™). Both techniques showed accuracy and reproducibility for indel frequencies across mosquito samples containing different ratios of indels of various sizes. Moreover, these techniques have advantages that make them potentially better suited for high-throughput nonhomologous end joining analysis in cage trials and contained field testing of gene-drive mosquitoes.

## METHOD SUMMARY

Mosquito DNA was extracted with the Promega Wizard^®^ Genomic DNA Purification Kit protocol and quantified with Qubit^®^ 3.0 following manufacturer protocols. PCR products for IDAA and ddPCR were generated with primers spanning 150–500 bp around the target site. IDAA amplicons were sent directly to COBO Technologies for analysis. ddPCR amplicons were analyzed using the Bio-Rad QX200™ ddPCR system.

CRISPR/Cas9 gene-editing technology has transformed the field of genome modification. This system is composed of two fundamental components that interact to form a complex: Cas9 endonuclease and sgRNA, a target-specific RNA that guides Cas9 to the desired genomic DNA target site. Cas9 induces a double strand break at the target site, activating the DNA repair pathways of homology-directed repair (HDR) and nonhomologous end joining (NHEJ). HDR can induce accurate gene repair of one to thousands of base pairs in the presence of a homologous donor molecule, allowing for the correction of point mutations and introduction of exogenous sequences. In contrast, NHEJ produces genetic lesions comprised of random sizes of small insertions or deletions (indels) that alter the target site and can disrupt gene function. The HDR mechanism offers the opportunity to genetically modify large populations of arthropods, among other model organisms, by integrating the *Cas9* endonuclease gene, the sgRNA targeting the desired locus and a dominant marker (fluorescent protein). The cassette is autonomous and can replicate to the homologous chromosome through HDR. This process effectively converts a heterozygous organism into a homozygote for the desired synthetic cassette, resulting in a selfish pattern of inheritance [1]. The nature of this type of genetic modification is designated gene drive and has been proposed as a tool for genetically modifying mosquito populations [2,3].

Gene drive in mosquitoes has been proposed as a promising tool for combating malaria and other mosquito-borne diseases, including dengue and zika [4], either by population suppression by spreading a lethal gene in wild-type (WT) mosquito populations to cause population crash or by replacement through the introduction of an anti-pathogen gene into a WT population. Recent progress demonstrated that CRISPR/Cas9 gene-drive-derived systems drive target-specific gene conversion at ≥99.5% efficiency in transgene heterozygotes of the *Anopheles stephensi* AsMCRkh2 line [5]. Gene drive efficiency depends on the availability of WT or susceptible alleles targeted by the gRNA-directed Cas9 cleavage. When a susceptible chromosome has been mutated by NHEJ, the key nucleotides necessary for gRNA recognition could be mutated or eliminated, thus preventing subsequent HDR-mediated gene conversion in the mosquito germline. An accumulation of NHEJ events has a diminishing effect on the drive, and the mosquito progeny approach Mendelian inheritance of the introduced DNAs due to the generation of drive-resistant loci [5,6]. Methods to detect NHEJ events rely on artificial reporter assays, gel-based systems, Sanger sequencing and deep sequencing [7–9]. None of these methods is suitable for high-throughput screening of NHEJ alleles in samples from multiple, large-cage populations or field trials due to their technical complexity, cost and time or labor required. A resistant Cas9-induced NHEJ allele percentage is considered acceptable when it is lower than the naturally occurring single nucleotide polymorphisms (SNPs) at the target site in the wild population [10]. This percentage can be tolerated while not affecting drive fixation; therefore, NHEJ quantification is an essential parameter during laboratory and field trials. Detecting indels in large populations of mosquitoes over many generations requires a high-throughput method that maximizes efficiency and provides sensitive, accurate results. To circumvent the difficulties of conventional techniques, we compared two novel techniques, Droplet Digital PCR™ (ddPCR™; Bio-Rad Laboratories, CA, USA) and Indel Detection by Amplicon Analysis (IDAA™; COBO Technologies, Copenhagen, Denmark) for NHEJ quantification in the *A. stephensi* AsMCRkh2 line carrying a CRISPR/Cas9 gene drive.

## Materials & methods

### Sample sources

*A. stephensi* mosquitoes (Indian strain, gift of M. Jacobs-Lorena, Johns Hopkins University) maintained at the University of California, Irvine (UCI) insectary are the source of all insects used in the experiments. The gene-drive line AsMCRkh2 (gene drive) and WT (non-gene drive) mosquitoes were maintained at 27°C with 77% humidity and a 12-h day/night, 30-min dusk/dawn lighting cycle. AsMCRkh2 mosquitoes with indels were recovered from crosses between WT and AsMCRkh2 mosquitoes over 20 generations [11]. The Cas9-targeted sequence, 5′- GATGGTTCCGTTCTACGGGCAGG-3′ (protospacer adjacent motif sequence underlined), is in the gene encoding *kynurenine hydroxylase* (*kh*).

### DNA extraction & quantification

Genomic DNA extraction was performed using the Wizard^®^ Genomic DNA Purification Kit protocol (Promega, WI, USA) for mouse tails according to the manufacturer’s instructions. Pools of 10 adult mosquitoes were used for DNA extraction. DNA was resuspended in 50 μl of PCR-grade water. DNA extracts were quantified at the UCI Genomics High-Throughput Facility using a Qubit^®^ 3.0 Fluorometer (Thermo Fisher Scientific, MA, USA) following the manufacturer’s instructions. One microliter of DNA extract was analyzed using the Qubit dsDNA HS Assay Kit followed by Qubit 3.0 quantification.

### ddPCR drop-off assay

We prepared 25-μl reactions with 12.5 μl Bio-Rad ddPCR 2× Supermix for Probes (No dUTP), 10 μl DNA (0.9 ng/μl), 1.25 μl fluorescein amidite (FAM)/forward (5-μM FAM probe, 18-μM forward primer) (Supplementary Table 1) and 1.25 μl hexachlorofluorescein (HEX)/reverse (5 μM HEX probe, 18-μM reverse primer) (Supplementary Table 1) in a 96-well PCR plate. Twenty microliters from the PCR reactions were used for droplet generation, each theoretically containing 30,000 haploid genome copies per 20-μl reaction, assuming that one *A. stephensi* haploid genome is 0.24 pg [12]. Droplets were generated at the UCI Genomics High-Throughput Facility using a Bio-Rad QX200 Droplet Generator following the manufacturer’s instructions; they were then transferred to a Bio-Rad 96-well PCR plate and foil heat-sealed at 180°C for 5 s. PCR was performed using a Bio-Rad C1000 Touch™ thermal cycler with a 96-deep-well reaction module under the following conditions: 95°C for 10 min, 40 cycles of 94°C for 30 s, 55°C for 1 min and 60°C for 2 min, followed by 98°C for 10 min and a 4°C hold. A 2°C/s ramp rate was used for all steps. Droplets were read using the Bio-Rad QX200 ddPCR system. The data analysis was performed using Bio-Rad QuantaSoft™ Analysis Pro version 1.0.596 in drop-off mode requiring manual cluster designation.

### IDAA assay

Samples were prepared from 25-μl PCR reactions using 0.5 U of TEMPase (Amplicon, Odense, Denmark) in 1× ammonium buffer with 2.5-mM MgCl_2_, 200-μM dNTP, 5% DMSO, 0.25-μM Universal FamFor, 0.025-μM forward-extension primer and 0.25-μM reverse-extension primer (Supplementary Table 1). PCR conditions included an initial incubation at 95°C for 15 min; 15 cycles of 95°C for 30 s, 72°C for 30 s and 72°C for 30 s, with the annealing temperature decreasing 1°C per cycle beginning from 72°C; and an additional 25 cycles of 95°C for 30 s, 58°C for 30 s and 72°C for 30 s, with 7 min of final extension at 72°C. PCR products were run in 3% agarose gel and analyzed directly. Samples were sent to COBO Technologies for fragment analysis and Profile IT Solutions (New Delhi, India) indel profiling and quantification.

## Results & discussion

**Figure 1.**
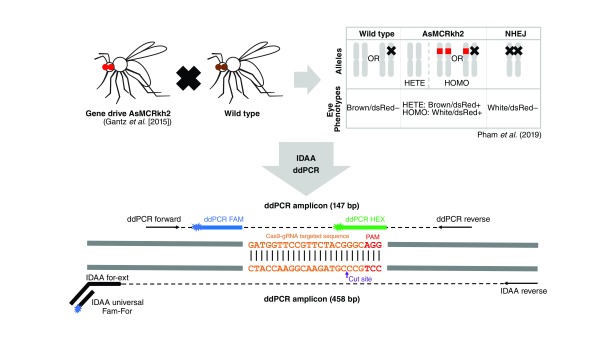
Assessment of nonhomologous end joining alleles from AsMCRkh2 mosquitoes using ddPCR and IDAA techniques. AsMCRkh2 is a gene drive transgenic *Anopheles stephensi* mosquito line that contains an autonomous Cas9-gRNA system linked to a dominant DsRed eye marker that targets the *kynurenine hydroxylase* (*kh*) locus [5]. WT mosquitoes and heterozygous AsMCRkh2 mosquitoes with one intact *kh* allele have a black-eyed/DsRed-positive phenotype, whereas homozygous AsMCRkh2 individuals have a recessive white-eyed/DsRed-positive phenotype. In contrast, mosquitoes presenting both resistant alleles present a white-eyed/DsRed-negative phenotype. NHEJ mosquitoes used in this report came from cage trials established from an outcross of AsMCRkh2 mosquitoes with WT where a susceptible *kh* allele was cleaved by gRNA-guided Cas9 nuclease and was repaired with NHEJ instead of HDR [5,11] to become a Cas9-resistant *kh* allele with mutations around the cut site. NHEJ mosquitoes from different cage generations were used for DNA extraction and PCR using primers designed to amplify a 458-bp PCR fragment and 147-bp PRC fragment spanning the targeted sequence for IDAA and ddPCR analysis, respectively. ddPCR: Droplet Digital PCR; HDR: Homology-directed repair; IDAA: Indel detection by amplicon analysis; NHEJ: Nonhomologous end joining; WT: Wild-type.

ddPCR is based on mechanically emulsifying a PCR solution into thousands of nanoliter droplets, effectively monitoring thousands of PCR reactions individually and thereby vastly increasing accuracy and reproducibility. It utilizes two fluorescent probes to discern WT and indel sequences: a HEX probe targets the WT gRNA site, and a FAM probe targets a conserved sequence within the amplicon (Figure 1; Supplementary Table 1). Sequences that are WT will give a fluorescent signal for both probes, and sequences possessing indels at the gRNA cut site display only a FAM signal, with the HEX probe failing to anneal. Each PCR-amplified nanoliter droplet is measured for these fluorescent signals, allowing statistically powerful quantification of indels present in PCR reactions. The alternative technique, IDAA, utilizes triple-primer PCR amplification to fluorescently tag the amplicon that includes the gRNA target site (Figure 1) [13]. Amplicons have their base-pair length measured by capillary gel electrophoresis; the WT length is used as a standard, and sequence lengths that differ are designated indels. The fluorescent signal allows unbiased quantification of amplicons, and the indel size is capable of being determined, importantly, without dependence on prior knowledge of the nature of the indels induced after Cas9:gRNA targeting.

IDAA and ddPCR were tested with a variety of indel mosquito samples to verify their sensitivity toward multiple mutations at the target sites (Figure 1). We examined mosquito samples obtained from a series of small-cage trials of the gene drive AsMCRkh2 strain of the Asian malaria vector mosquito, *A. stephensi* [5,11]. We analyzed 15 pools of 10 mosquitoes each that were considered to have a NHEJ by phenotype selection (white-eye and DsRed-negative) based on previous data [5]. However, because the *kh* mutant white-eye phenotype is associated with a recessive mutation, no phenotypic selection was possible until the second generation (G2). Previous work with these NHEJ mosquitoes had shown that Cas9 indel mutations happened at and around the cut site and protospacer adjacent motif sequence, resulting in insertions and deletions of multiple lengths ranging from 1 to 473 bp [11]. With 185 NHEJ individuals analyzed by Sanger sequencing, 50 different types of indels were identified, including three types of 1-bp indels (from 15 individuals, ∼8% of 185) and one type of substitution (from 1 individual, ∼0.5%) [11]. Two sets of samples were generated: NHEJ samples, which contained only pools of confirmed NHEJ individuals obtained from previous cage experiments with white-eye and DsRed-negative phenotypes to challenge the sensitivity of the two techniques toward different types of NHEJ, and mixed samples, which were generated by using DNA extracted from a mixture of NHEJ mosquito samples with WT mosquitoes at different proportions to quantify the NHEJ proportion in those samples.

Results from both IDAA and ddPCR experiments for the NHEJ samples showed that both techniques were able to detect all mutant sequences in the NHEJ mosquito samples with an indel percentage of 100%. All samples were analyzed by three technical replicates, with the average total percentage of indels shown in Table 1 and Supplementary Table 2. Both IDAA and ddPCR provide a quantitative analysis of total indel percentage, but only IDAA details the indel sizes and their respective proportions in a sample. Based on the IDAA analysis, indels were detected in a range from 1 to 469 bp, including insertions and deletions, thus representing a broad variety of Cas9-induced indels (Table 2). Many samples contained multiple different indels that were quantified for the proportional amount of each indel present in the sample. Sequencing data for some chosen NHEJ individuals are listed in Supplementary Table 3, and these confirmed the sensitivity accuracy of IDAA and ddPCR for different types of indels. Not all indels were identified with Sanger sequencing because of the time and labor costs necessary to extract and sequence all individuals, limited sources of genomic DNA from single mosquito extractions and PCR technical problems. In addition, not all samples were suitable for Sanger sequencing because DNA was extracted from mosquito pools and included a mixture of mutations. This level of complexity reduced the reliability of PCR amplification because not all of the mutations could be amplified with the same efficiency due to variants of different indel frequencies, resulting in nonspecific sequencing errors. Providing sequencing data for each sample via next-generation sequencing would costly, unnecessary and difficult due to the abovementioned reasons regarding the quality of DNA extracts and amplification of different indels in a pooled sample.

**Table 1.** Insertions or deletions quantification in nonhomologous end joining mosquito samples from small-cage trials of the gene drive AsMCRkh2 strain.

Number	Sample (cage name-generation)	ddPCR average indel (%)	IDAA average indel (%)
Indel-1	A1-G3	100.00	100.00
Indel-2	A1-G8	99.97	100.00
Indel-3	A1-G14	100.00	100.00
Indel-4	A1-G16	100.00	100.00
Indel-5	A3-G4	100.00	100.00
Indel-6	A3-G7	100.00	100.00
Indel-7	A3-G8	100.00	100.00
Indel-8	A3-G9	100.00	99.20
Indel-9	A3-G10	100.00	100.00
Indel-10	B1-G4	100.00	100.00
Indel-11	B1-G7	99.97	100.00
Indel-12	B1-G9	100.00	100.00
Indel-13	B1-G10	99.80	100.00
Indel-14	C1-G8	100.00	100.00
Indel-15	C1-G11	99.97	100.00

A total of 15 sample pools of ten mosquitoes each were obtained from different cages through several generations [11]. ddPCR and IDAA were used to analyze the same DNA extract of each sample to quantify the total percentage of indel sequences. Analysis was carried out in triplicate (n = 3) with averages shown. The Pearson correlation coefficient is *r* = 0.77 when comparing similarity trends. Student’s *t* tests performed for each individual sample yielded no statistical significance (p < 0.05) between the results of both techniques. IDAA and ddPCR are sensitive in detecting multiple types of indels in a pool sample, as there is no significant difference between the results of the two methods and the expected percentage of indel, which is 100% in all samples. Cage numbers refer to those in Pham *et al.* [11].

ddPCR: Droplet Digital PCR; IDAA: Indel detection by amplicon analysis; Indel: Insertions or deletion.

**Table 2.** Insertions or deletion lengths detected by IDAA.

Indel source	Frameshift indels (%)	Length (%)				
		First top indel	Second top indel	Third top indel	Fourth top indel	Fifth top indel
A1-G3	100	2 (52.8)	-2 (47.2)	—	—	—
A1-G8	100	-8 (61.9)	7 (27.0)	-11 (11.1)	—	—
A1-G14	42.8	-11 (34.5)	-3 (27.4)	-6 (20.1)	18 (9.7)	8 (8.3)
A1-G16	100	-13 (85.3)	469 (14.7)	—	—	—
A3-G4	51.2	-48 (48.8)	11 (22.0)	-13 (17.5)	8 (7.3)	-49 (4.5)
A3-G7	100	5 (54.0)	8 (46.0)	—	—	—
A3-G8	100	1 (62.4)	11 (37.6)	—	—	—
A3-G9	53.9	-33 (46.1)	11 (44.7)	1 (4.9)	-34 (4.3)	—
A3-G10	32.1	-33 (65.5)	1 (26.2)	-34 (6.0)	—	—
B1-G4	14.1	-6 (85.9)	-7 (13.0)	-29 (1.1)	—	—
B1-G7	100	-4 (51.5)	1 (27.5)	-14 (11.5)	8 (9.5)	—
B1-G9	100	-4 (54.7)	1 (39.9)	-5 (5.5)	—	—
B1-G10	100	-4 (90.7)	-5 (9.3)	—	—	—
C1-G8	100	-10 (51.6)	2 (20.0)	2 (19.0)	-14 (9.5)	—
C1-G11	100	-4 (90.4)	-5 (9.6)	—	—	—

IDAA allows quantification of each indel sequence of different length, giving insight into the indel composition of the DNA extract. IDAA analysis was done in triplicate (n = 3) with the averages of the top five most prevalent indel lengths displayed from each sample source, the same source used for the analysis detailed in Table 1. Total frameshift indel percentages are provided by excluding indel sizes that are divisible by three. Indels found across all samples range from deletions of 48 bp to insertions as large as 469 bp. Identified mutations included insertions (+) and deletions (-) with different lengths as small as 1 bp. The percentage of each indel is shown in parentheses.

ddPCR: Droplet Digital PCR; IDAA: Indel detection by amplicon analysis; Indel: Insertions or deletion.

Sensitivity and quantification of 1-bp insertions by IDAA can be seen in samples G8A3, G9A3, G10A3, G7B1 and G9B1 (Table 2 & Supplementary Table 3). The same DNA extracts from all samples were used for both techniques, allowing a direct comparison of indel quantification. Because the ddPCR technique designated the same sample extracts at or near 100% indel, it demonstrates that the 1-bp insertions in those samples are being reliably detected (Table 1). This is consistent with prior data supporting the 1-bp indel sensitivity of both IDAA and ddPCR [13,14]. Overall, every indel size discovered by the IDAA method was detected by ddPCR, as it designated all samples as 100% or near 100% indel with no significant differences observed between individual samples and a strong correlation coefficient of 0.73 (Table 1). If ddPCR were insensitive to a certain indel identified in a sample by IDAA, then the total indel percent determined by ddPCR would proportionally reflect an increase in WT percentage. Samples G8A1, G9A3, G7B1, G10B1 and G11C1 were slightly below 100% indel frequency in either technique, and this is likely due to fluorescence anomalies. The nominal WT sequence quantified in these samples (0.03–0.8%) is unreliable because, at its lowest frequency, a true WT allele in a pool of ten indel mosquitoes (20 alleles total) would produce a 5% WT (or 95% indel) proportion, which was not observed.

**Figure 2.**
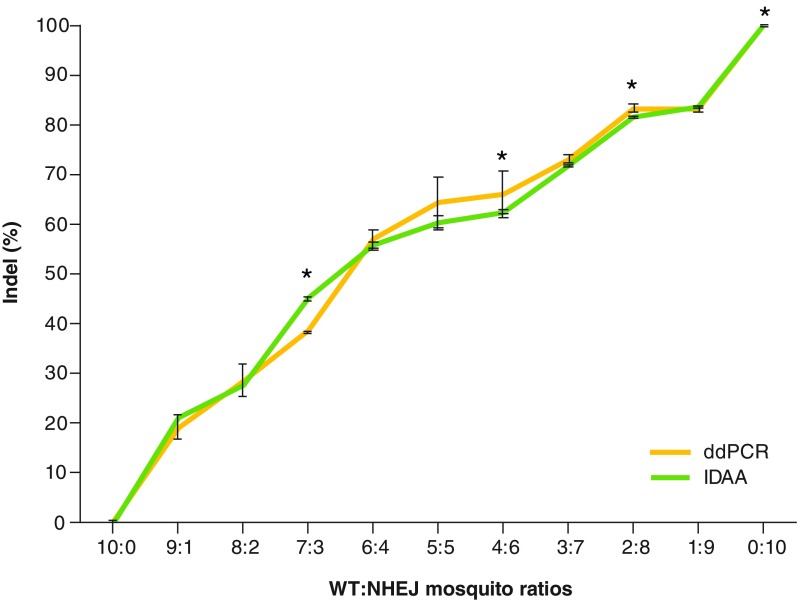
Quantification of nonhomologous end joining alleles in AsMCRkh2 mosquito samples by ddPCR and IDAA techniques. DNA was extracted from 15 to 10 mosquito pools. To assess the sensitivity of both techniques, the mosquito pools consisted of a mix of WT and NHEJ mosquitoes at 11 different ratios of WT:NHEJ (10:0, 9:1, 8:2, 7:3, 6:4, 5:5, 4:6, 3:7, 2:8, 1:9 and 0:10). DNA was used for PCR, and amplicons were subjected to IDAA and ddPCR analysis to determine the indel percentage in each sample. Each ratio was conducted in triplicate (n = 3), and average results were compared between the two techniques. Although deviating from theoretical indel percentages (40% indel for a 6:4 ratio), both techniques demonstrated precision based on producing similar results for each ratio and having a Pearson correlation coefficient of *r* = 0.99. In addition, values provided by ddPCR and IDAA are also representative of their accuracy; because both deduced the same indel percentage, it is likely close to the actual indel percentage. Student's *t* test was performed to compare the measurements at each ratio (*p < 0.05). ddPCR: Droplet Digital PCR; IDAA: Indel detection by amplicon analysis; Indel: Insertions or deletion; NHEJ: Nonhomologous end joining; WT: Wild-type.

**Figure 3.**
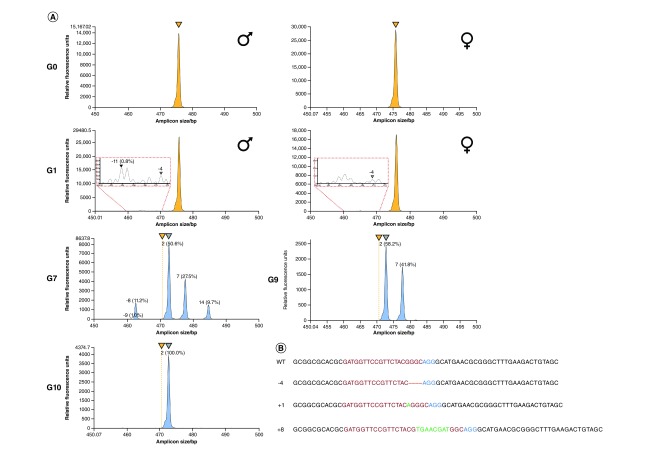
Tracing and quantification of insertions or deletions in AsMCRkh2 mosquito samples over generations. G0 and G1 mosquito samples were chosen randomly, and samples from G7 through G10 were all individuals carrying NHEJ alleles selected by phenotype (white-eyed/DsRed-negative). **(A)** G0: ‘Founder’ individuals show the baseline for the IDAA profile. Both males and females present a WT sequence at the target site shown by a yellow peak (because all female G0s are WT, whereas even though G0 males were a combination of transgenic and WT males only WT [and NHEJ alleles, if there were any] were amplified by PCR). G1: First-generation offspring display expected low frequency of indels in sample pools of WT and low-frequency NHEJ individuals. Red-dotted line zoom-in inserts display the rarely occurring NHEJ indel events in the population (<0.8%), and the black and gray triangles indicate spectra peaks of indels. Two types of indels, -11 and -4, were identified in G1. IDAA and ddPCR allowed the analysis of a large number of samples from G0 and G1, which was required to determine NHEJ allele-generated frequency. G7–G10: White-eyed phenotype mosquitoes with homozygous NHEJ alleles were selected from different generations. Although phenotypically similar, the variable peak heights indicate that G7 individuals represent a heterogeneous population, with different types of indels, whereas G9 (near equal peak heights) and G10 (single peak) represent a homogeneous population with only one indel (-4) selectively carried to subsequence generations. WT alleles are distinguished by yellow peaks when present in the spectra; when absent, yellow triangles above the spectra panels are used to reference the WT location. Frameshift-causing indels are indicated with peaks color-coded in blue. **(B)** Sanger sequencing in mosquitoes from G7 (11 individuals), G9 (5 individuals) and G10 (16 individuals) show results comparable with the IDAA findings. Three types of indels (-4, +1, +8) were identified in G7 mosquitoes, whereas only one type of indel (-4) was present in G9 and G10 (Supplementary Table 3). ddPCR: Droplet Digital PCR; IDAA: Indel detection by amplicon analysis; Indel: Insertions or deletion; NHEJ: Nonhomologous end joining; WT: Wild-type.

In order to assess accuracy and replicate a trial scenario in which quantification techniques are employed, 11 pooled samples of NHEJ mosquitoes were made with WT mosquitoes at different ratios of WT:NHEJ mosquitoes (10:0, 9:1, 8:2, 7:3, 6:4, 5:5, 4:6, 3:7, 2:8, 1:9 and 0:10) within a pool of 10 total mosquitoes (Figure 2 & Table 2). In the mixture of NHEJ mosquitoes from the A3 to G4A3-G4 samples with multiple types of mutations, both ddPCR and IDAA techniques showed indel frequencies similar to theoretical frequencies (e.g., 5:5 ratio sample produces 50% indel and 50% WT), as well as both techniques having similar percentages, and no statistical differences were observed for the majority of samples. Also, a similar trend in the deviation from theoretical frequencies can be observed in both techniques as supported by a correlation coefficient of 0.99 (Figure 2). Statistically significant differences were observed for the 7:3, 4:6, 2:8 and 10:0 ratios between the IDAA and ddPCR percentages, which may be due to the detection or binding of fluorescent probes/primers and unequal amplification during PCR processes. Moreover, IDAA allows the identification of indel sizes, enabling the approach of tracking an indel as it is inherited through multiple generations as previously shown when indel germline transmission rates were traced in zebrafish [15]. Samples from different mosquito generations of the same cage population can be used to identify multiple indels across several generations (-4, 1 and -5) (Figure 3A). Randomly chosen individuals analyzed with Sanger sequencing confirmed the results obtained by IDAA and ddPCR and showed that the detected indels are accurate (Figure 3B). Some indels were identified by IDAA but were not identified with Sanger sequencing (-16 in G7 and +1 in G9), indicating that IDAA allows a broader coverage of analysis and prevents missing important indels due to small samples size as when analyzed by Sanger sequencing.

Both IDAA and ddPCR have beneficial characteristics beyond their technical capabilities, including the cost and timeline for acquiring large datasets. The exact cost of these techniques for a project is difficult to compare because the prices for services vary among institutions and depending on where the techniques are sourced. An estimation of the total cost per sample for either ddPCR or IDAA is around $20. Reagent costs vary based on the amount purchased, but become negligible compared with analysis costs in a large experiment. For our purposes, both IDAA and ddPCR had comparable costs of reagents, with the latter having lower operational costs due to being performed at a nonprofit UCI facility. If instrumentation is at hand, the workbench procedure for IDAA, PCR amplification using the triple-primer PCR protocol, can be completed within a day [16]. Subsequently, the samples can be shipped to COBO Technologies for analysis, and the results can be obtained in less than a week, or within days in ‘fast track’ mode, after samples are received. Samples for ddPCR can be fully prepped, assayed and analyzed in a single day if instrumentation is available.

Both methods are far more cost effective than deep-sequencing techniques. For example, the Illumina MiSeq™ System (CA, USA) platform price is approximately $400 to run a variable number of reactions and an additional $95 per reaction for library preparation. Moreover, analysis of deep sequencing data requires significant bioinformatics expertise, which is not required for either IDAA or ddPCR. High-resolution melting analysis is another cost-effective, viable option for mutation analysis and genotyping but lacks the quantification capabilities of ddPCR and IDAA. The detectable threshold is higher for high-resolution melting analysis at 10%, whereas IDAA and ddPCR are more sensitive, detecting mutant sequences as low as 0.1% [17]. Although IDAA is capable of providing more information on indel sizes and relative composition, most of the indels are observed within ±20 bp from the putative double strand DNA break site in another mosquito species (*Aedes aegypti*); therefore, ddPCR and IDAA cannot detect any large deletions beyond the window of ±20 bp [18]. Assay wipeout can occur for ddPCR if a deletion is large enough to disrupt the sequence of the reference probe (FAM) binding site; in this case, no probes are bound, resulting in a false-negative signal. Guidelines specify that the reference probe should be at least 25 bps from the gRNA cut site to prevent this, so, depending on the distance used in assay design, the potential for this occurrence is variable.

In these comparative experiments, we demonstrated that ddPCR and IDAA are promising techniques for the quantification and analysis of NHEJ alleles in gene drive mosquitoes. These techniques showed sensitive and reproducible detection of NHEJ events and can be used instead of next-generation sequencing for a high-throughput protocol that saves time and reduces cost. This approach offers a more efficient analysis of gene-drive cage experiments and field trials where quantification of NHEJ is important as an indicator of potential resistance alleles that can prevent complete drive introduction into field populations [11,19]. Both techniques have their strengths and weakness depending on the purposes of the user. Because IDAA detects indels uniquely by length deviations, the technique will overlook point mutations such as substitutions, which were rare events in mosquito gene-drive systems [4,5,11]. In addition, highly variable or nonconserved DNA regions may not be suitable for IDAA analysis because of the presence of preoccurring indels that interfere with the detection of NHEJ-induced indels. Because IDAA cannot detect SNPs and the current application is to quantify NHEJ-induced indels, the analysis of SNPs in this experiment was omitted, although ddPCR had been shown to detect SNPs as mutant sequences [20]. However, IDAA provides the percentage of each different indel in a mixture, and this information can be useful for tracking mutations through successive generations in a cage trial format or in open release trials as a surveillance approach. In contrast, ddPCR identifies mutations by binding of probes at the target site and thus has greater sensitivity for all types of indels. The detection of SNPs could potentially interfere with a NHEJ quantification assay, due to the same observable output between SNPs and indels (failure of gRNA cut site probe to anneal). A benefit of ddPCR is that the equipment can be easily transported, which is suitable for analyzing gene drive efficiency in the field where resources for sample prep, shipment and analysis are limited. Unlike IDAA, ddPCR can detect substitution mutations but is not effective for tracking indels over generations. Potentially the largest drawback shared by both techniques is the lack of sequence data, given such information is pertinent for answering the research question. In the case of a screening application where samples with indels are rare, ddPCR or IDAA can be coupled with sequencing to acquire sequence data while maintaining high-throughput efficiency.

IDAA and ddPCR showed sensitive and reproducible detection of NHEJ events in mosquito samples from cage experiments. Both techniques offer a more efficient analysis of indel quantification in a cost- and time-saving manner, and they can be used for efficient analysis of gene-drive mosquito populations for quantifying NHEJ. Thus, they possess the qualifications to determine factors that will influence gene drive in cage trials or field releases.

## Future perspective

As CRISPR-Cas9-based gene drives are being widely developed for applications in vector control, ecology conservation and pest management, cage trials and field trials will likely become regulatory checkpoints for deploying gene drives in living organisms into the field. A high-throughput yet cost-effective method to determine NHEJ alleles compared with HDR for drive efficiency is necessary for the study of gene-drive behaviors in big population samples format.
