# Supplementary material for: Digital droplet PCR and IDAA for the detection of CRISPR indel edits in the malaria species Anopheles stephensi
Source: Biotechniques. 2020 Feb 10;68(4):172–9. doi: 10.2144/btn-2019-0103 (PMC7177198; doi:10.2144/btn-2019-0103)
Supplement: Supplementary file 1 [file btn-68-172-s1.docx]

**Table S1**. Sequences of primers and probes used in this study for ddPCR and IDAA experiments.

| **Primer/Probe** | **Sequence (5’ » 3’)** |
| --- | --- |
| ddPCR Forward Primer | ATGATCAAATGTCGACCG |
| ddPCR Reverse Primer | ACCGTACTGGTTGAACA |
| ddPCR HEX Probe (BHQ1) | [HEX]-TTCTACGGGCAGGGC-[BHQ1] |
| ddPCR FAM Probe (BHQ1) | [6FAM]-CCACGTGGGATCGAAGG-[BHQ1] |
| IDAA Forward-Extension Primer | AGCTGACCGGCAGCAAAATTGGCCAGACGTACATCGAGCACG |
| IDAA Reverse Primer | GTCCGTACCGTACTGGTTG |
| IDAA Universal FamFor | [6FAM]-AGCTGACCGGCAGCAAAATTG |

BHQ: Black Hole Quencher

**Table S2.** ddPCR and IDAA indel frequencies for sample replicates. Three replicates (A, B, C) used, average percentage used as final indel percentage.

|  | | | **ddPCR** | | | | **IDAA** | | | |  |
| --- | --- | --- | --- | --- | --- | --- | --- | --- | --- | --- | --- |
| **Samples** | **Indel % A** | **Indel % B** | | **Indel % C** | **Avg Indel %** | **Indel % A** | | **Indel % B** | **Indel % C** | **Avg Indel %** | |
| **10:0** | 0.61 | 0.34 | | 0.25 | 0.4 | 0.6 | | 0 | 0 | 0.22 | |
| **9:1** | 19.6 | 19.7 | | 18.8 | 19.37 | 19.1 | | 26.3 | 18.8 | 21.4 | |
| **8:2** | 28.8 | 28.5 | | 28.4 | 28.57 | 25 | | 26.9 | 31.4 | 27.77 | |
| **7:3** | 38.6 | 38.7 | | 38.5 | 38.6 | 44.8 | | 45.2 | 45.6 | 45.2 | |
| **6:4** | 56.4 | 58.1 | | 56.7 | 57.07 | 56.8 | | 54.9 | 55.4 | 55.7 | |
| **5:5** | 61.8 | 68.2 | | 63.4 | 64.47 | 62.1 | | 59.7 | 59.5 | 60.43 | |
| **4:6** | 65.3 | 66.1 | | 66.6 | 66 | 63 | | 62.7 | 62.1 | 62.6 | |
| **3:7** | 73.8 | 72.2 | | 73.6 | 73.2 | 72.2 | | 71.7 | 71.9 | 71.93 | |
| **2:8** | 82.9 | 83.6 | | 83.4 | 83.3 | 81.4 | | 81.9 | 81.4 | 81.57 | |
| **1:9** | 83.4 | 82.9 | | N/A | 83.15 | 83.6 | | 83.3 | N/A | 83.45 | |
| **0:10** | 99.9 | 99.9 | | 99.9 | 99.9 | 100 | | 100 | 100 | 100 | |
|  |  |  | |  |  |  | |  |  |  | |
| **A1-G3** | 100 | 100 | | 100 | 100 | 100 | | 100 | 100 | 100 | |
| **A1-G8** | 99.9 | 100 | | 100 | 99.97 | 100 | | 100 | 100 | 100 | |
| **A1-G14** | 100 | 100 | | 100 | 100 | 100 | | 100 | 100 | 100 | |
| **A1-G16** | 100 | 100 | | 100 | 100 | 100 | | 100 | 100 | 100 | |
| **A3-G4** | 100 | 100 | | 100 | 100 | 100 | | 100 | 100 | 100 | |
| **A3-G7** | 100 | 100 | | 100 | 100 | 100 | | 100 | 100 | 100 | |
| **A3-G8** | 100 | 100 | | 100 | 100 | 100 | | 100 | 100 | 100 | |
| **A3-G9** | 100 | 100 | | 100 | 100 | 100 | | 100 | 97.6 | 99.2 | |
| **A3-G10** | 100 | 100 | | 100 | 100 | 100 | | 100 | 100 | 100 | |
| **B1-G4** | 100 | 100 | | 100 | 100 | 100 | | 100 | 100 | 100 | |
| **B1-G7** | 99.9 | 100 | | 100 | 99.97 | 100 | | 100 | 100 | 100 | |
| **B1-G9** | 100 | 100 | | 100 | 100 | 100 | | 100 | 100 | 100 | |
| **B1-G10** | 99.9 | 99.8 | | 99.7 | 99.8 | 100 | | 100 | 100 | 100 | |
| **C1-G8** | 100 | 100 | | 100 | 100 | 100 | | 100 | 100 | 100 | |
| **C11-G11** | 99.9 | 100 | | 100 | 99.97 | 100 | | 100 | 100 | 100 | |

**Table S3:** Sanger sequencing data of NHEJ mosquitoes with indels identified by IDAA in Table 2.

| Cage | Generation | Sequence | Type of mutation |
| --- | --- | --- | --- |
| WT |  | **GCGGCGCACGCGATGGTTCCGTTCTACGGGCAGGGCATGAACGCGGGCTTTGAAGACTGTAGC** |  |
| A-1 | **G3** | **GCGGCGCACGCGATGGTTCCGTTCTACAGGGGCAGGGCATGAACGCGGGCTTTGAAGACTGTAGC** | +2 |
|  | **G3** | **GCGGCGCACGCGATGGTTCCGTTCTAC--GCAGGGCATGAACGCGGGCTTTGAAGACTGTAGC** | -2 |
|  | **G8** | **GCGGCGCACGCGATGGTTCCG--------GCAGGGCATGAACGCGGGCTTTGAAGACTGTAGC** | -8 |
|  | **G8,G14** | **GCGGCGCACGCGATGAAC-----------GCAGGGCATGAACGCGGGCTTTGAAGACTGTAGC** | -11 |
|  | **G14** | **GCGGCGCACGCGATGGTTCCGTTCCC---GCAGGGCATGAACGCGGGCTTTGAAGACTGTAGC** | -3 |
|  | **G16** | **GCGGCGCACGCGATGGTTCCGTTCTACG(biginsert)GGCAGGGCATGAACGCGGGCTTTGAAGACTGTAGC*** | +469 |
| A-3 | **G4** | **GCGGCGCACGCGATGGTTCCGTTCTACAGGAACGTTCAT-GGCAGGGCATGAACGCGGGCTTTGAAGACTGTAGC** | +11 |
|  | **G7** | **GCGGCGCACGCGATGGTTCCGTTCTACAAGGGAGGCAGGGCATGAACGCGGGCTTTGAAGACTGTAGC** | +5 |
|  | **G7** | **GCGGCGCACGCGATGGTTCCGTTCTACCATCATACAGGGCAGGGCATGAACGCGGGCTTTGAAGACTGTAGC** | +8 |
|  | **G8** | **GCGGCGCACGCGATGGTTCCGTTCTACGAGGCAGGGCATGAACGCGGGCTTTGAAGACTGTAGC** | +1 |
|  | **G8,G9** | **GCGGCGCACGCGATGGTTCCGTTCTACAAGGGAACAGAGGCAGGGCATGAACGCGGGCTTTGAAGACTGTAGC** | +11 |
|  | **G10** | **GCGGCGCAATCATCGGATCATCGGA---------------------------------GTAGC** | -33 |
| B-1 | **G4** | **GCGGCGCACGCGATGGTTCCGTTCTACA------GCATGAACGCGGGCTTTGAAGACTGTAGC** | -6 |
|  | **G7,G9,**  **G10** | **GCGGCGCACGCGATGGTTCCGTTCTAC----AGGGCATGAACGCGGGCTTTGAAGACTGTAGC** | -4 |
|  | **G7,G9** | **GCGGCGCACGCGATGGTTCCGTTCTACAGGGCAGGGCATGAACGCGGGCTTTGAAGACTGTAGC** | +1 |
| C-1 | **G11** | **GCGGCGCACGCGATGGTTCCGTTC----GGCAGGGCATGAACGCGGGCTTTGAAGACTGTAGC** | -4 |
|  | **G11** | **GCGGCGCACGCGATGGTTCCGTTCTAC----AGGGCATGAACGCGGGCTTTGAAGACTGTAGC** | -4 |
|  | **G8** | **GCGGCGCACGCGATGGTTCCGTTCTACCAT----------ACGCGGGCTTTGAAGACTGTAGC** | -10 |

**gRNA target site; PAM; insertion; deletion, substitution.**

***GGCGCACGCGATGGTTCCGTTCTACGGGCGTTTTAGAGCTAGAAATAGCAAGTTAAAATAAGGCTAGTCCGTTATCAACTTGAAAAGTGGCACCGAGTCGGTGCTTTTTTTGTGGAAATTTGATTCACTTGTTTTAGGAAAATAATTACCTTCCTTTGAAACAGGTATTTACAATGATGGACGATAGAAAAAGACCACCTTAAAATGCTTTATTCAAGCTATTGCTTTCGATTCTTCATTGAGATCTAATCTCAAATTTGTGTATTTAGAAAAAACAGCGAATATTGTACTATAATAATTCTTTGGTAATCTCAGGCTTTCAGTAATGATAGAATCCTTAAGACTGTAAGAATATACAAGTTTTAAAAAAAGGAAAACAGGTATATCAAATCAGAAAACAACAACAGCTCTTTTCCGTTGAATTCTACCTGTACGATGGCTTAAGGATGAGCTGTAACTGAAACGAAACCTTACAACAGCCACGCAGAACGGGATCCGGCAGGGCATGAACGC**

**Figures S1-26**. 2-D Fluorescent plot clusters for ddPCR analysis. Droplets were manually clustered according to fluorescent signals from FAM and HEX (WT, orange), FAM only (NHEJ, Blue), and negative (empty, gray). Plots displayed are a merger of the three replicates for each designated sample. Sample mixture ratios (WT:AsMCRkh2) are listed in top right of each panel (S1-S11), followed by the indel samples (S12-S26).


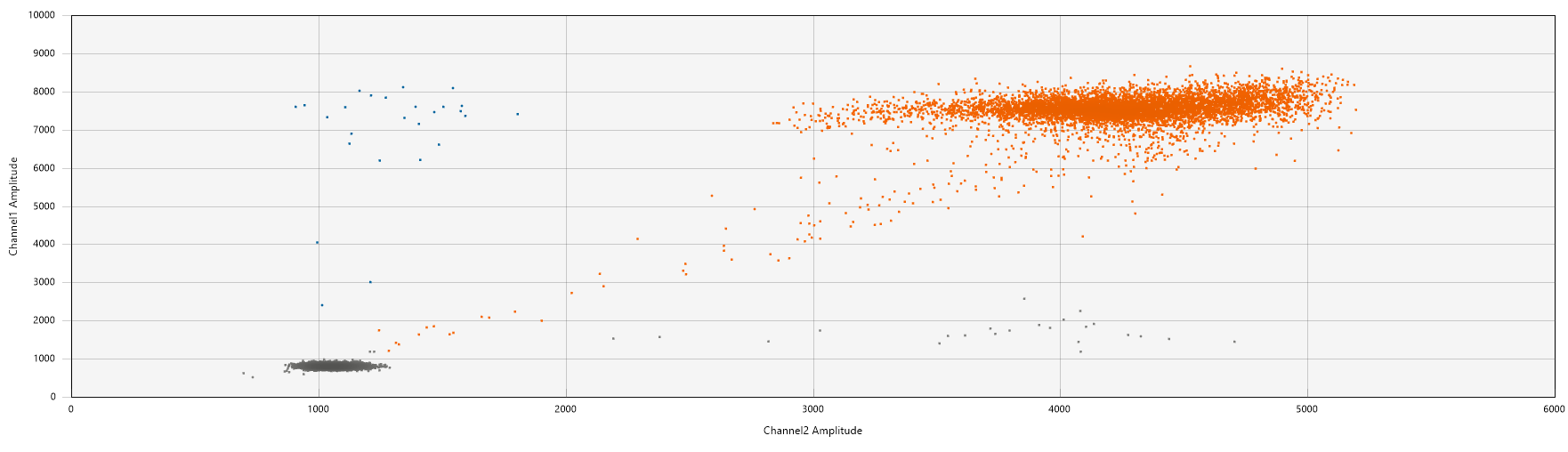

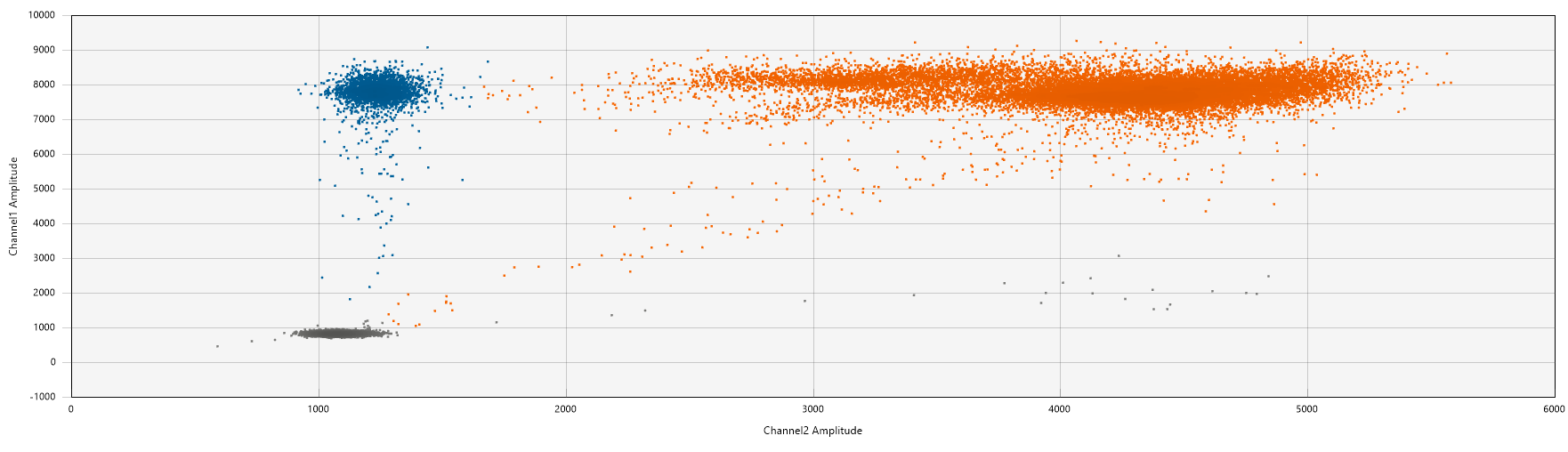

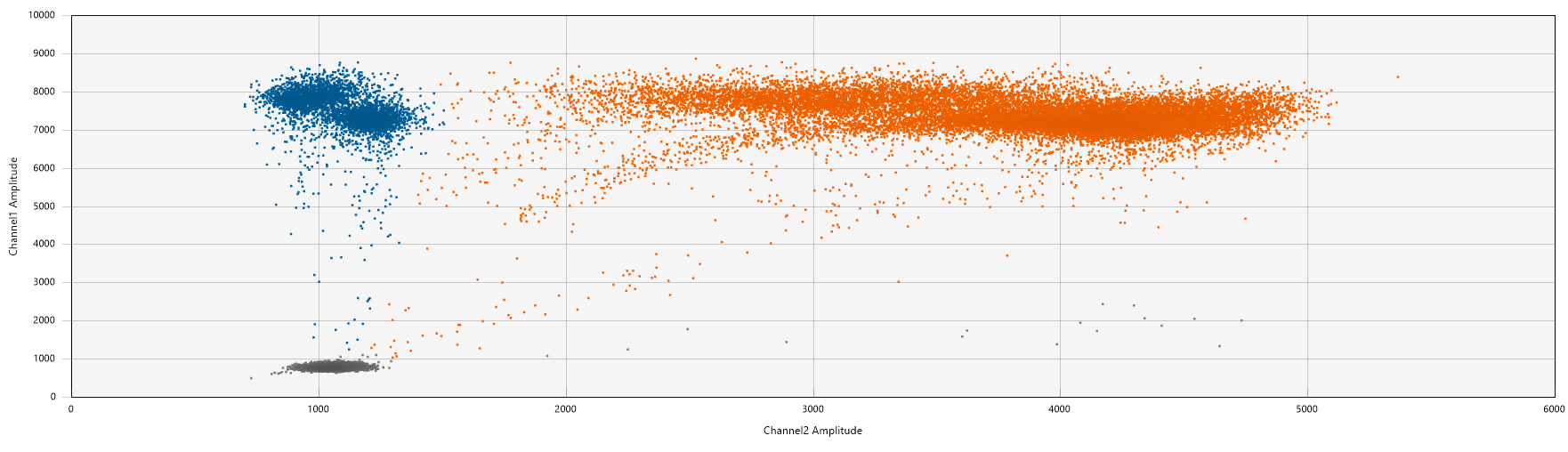

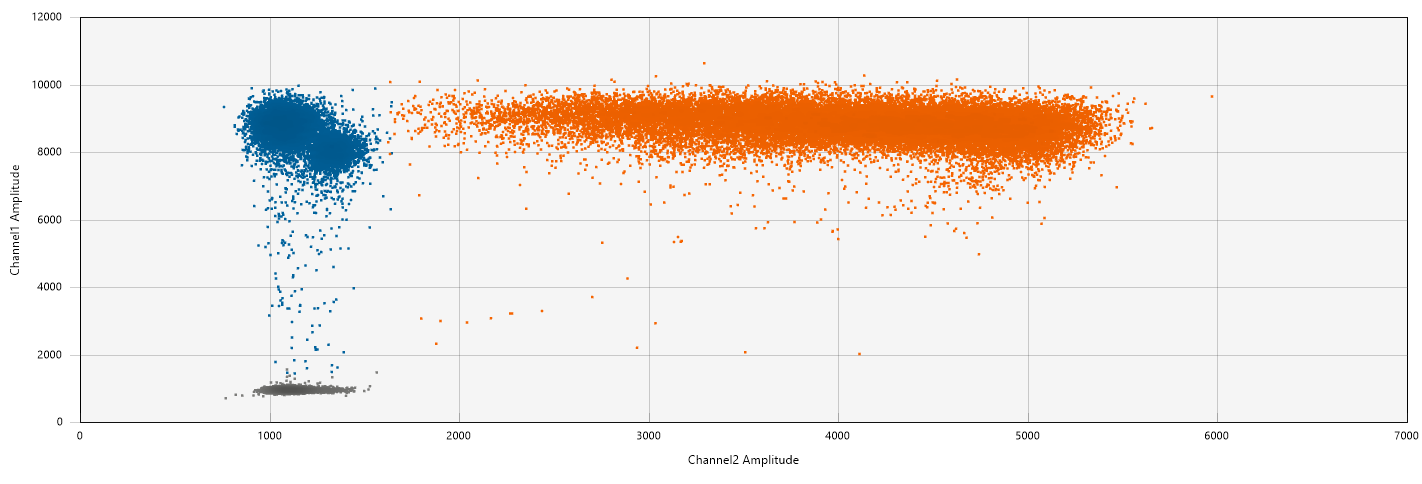

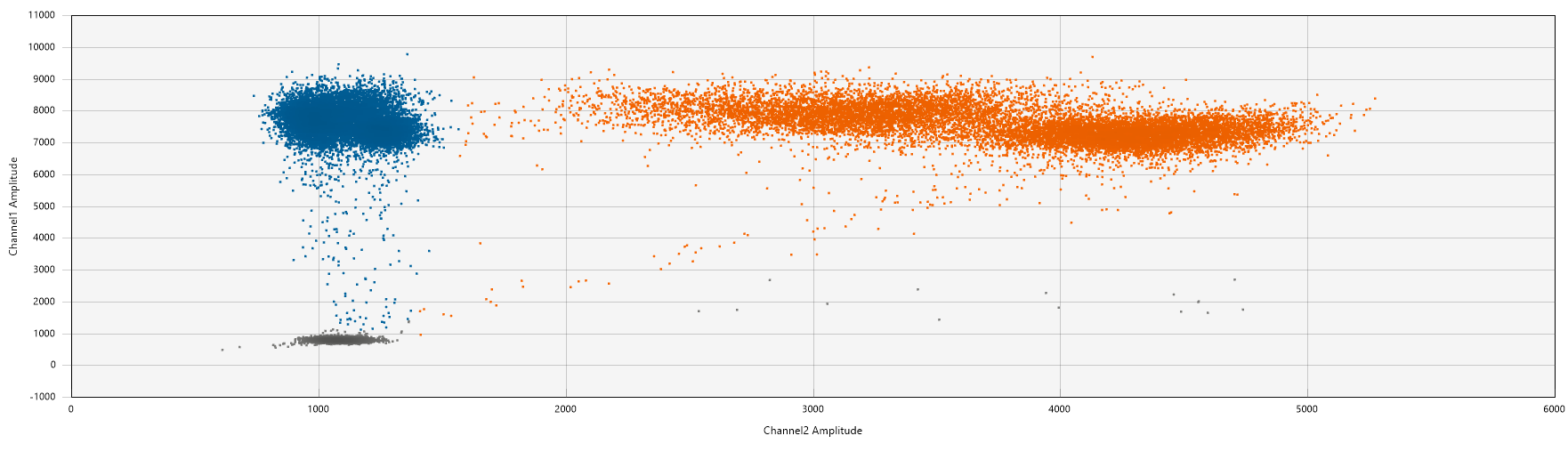

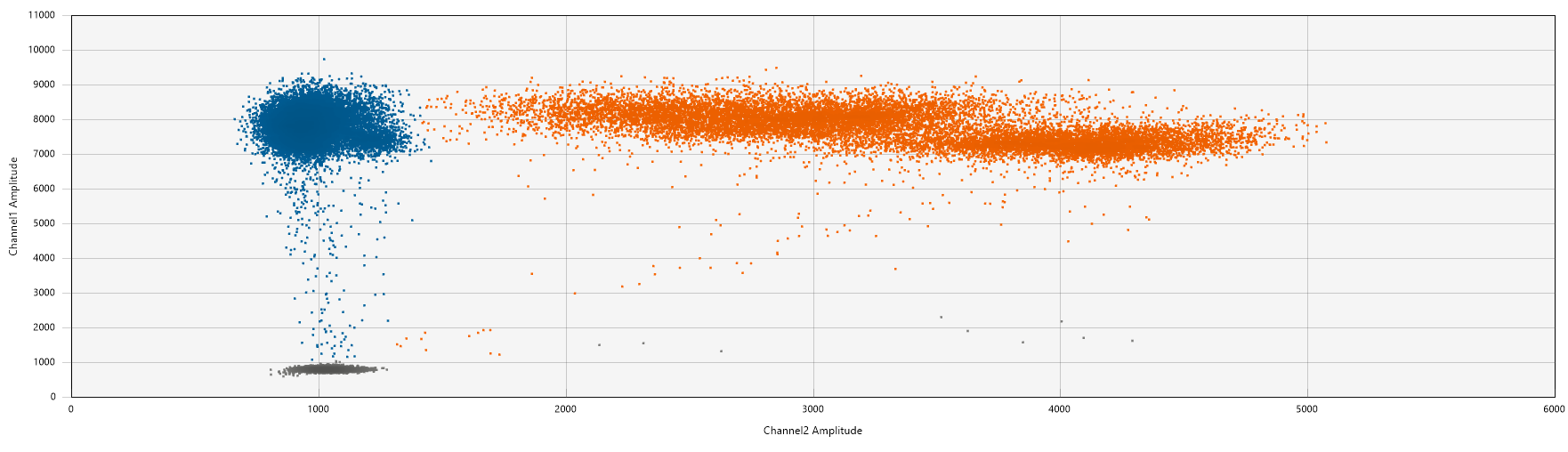

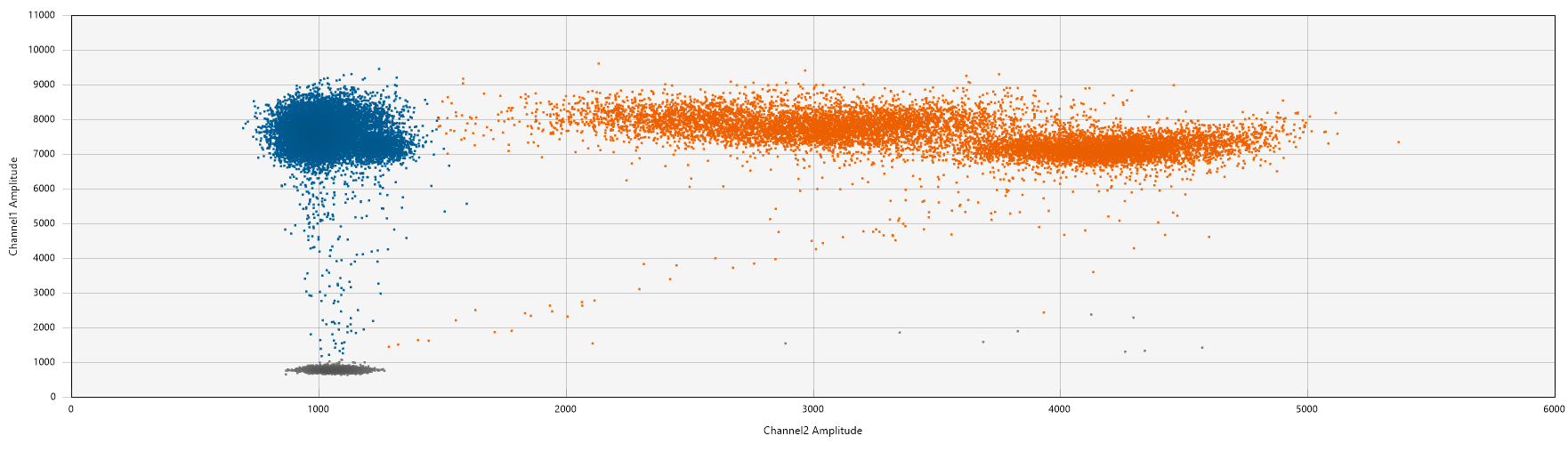

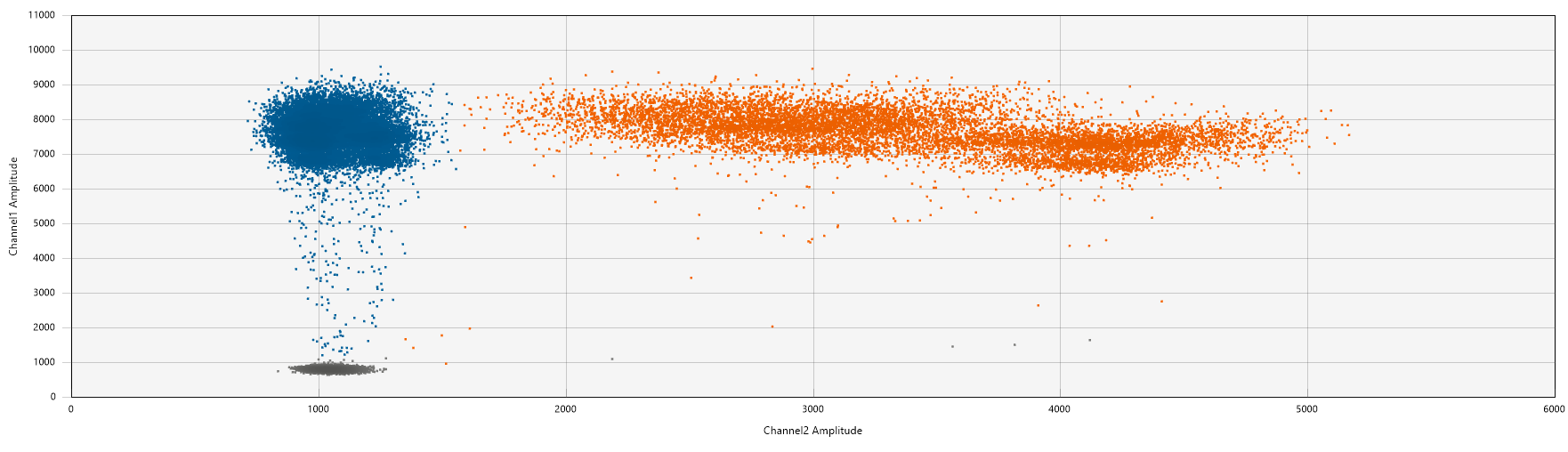

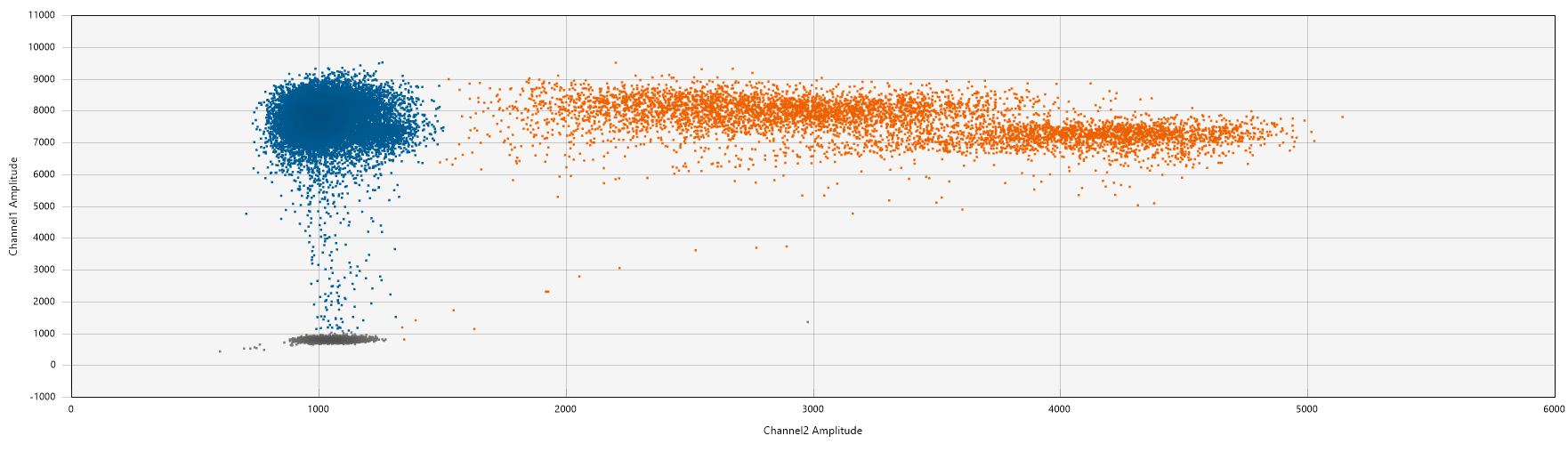

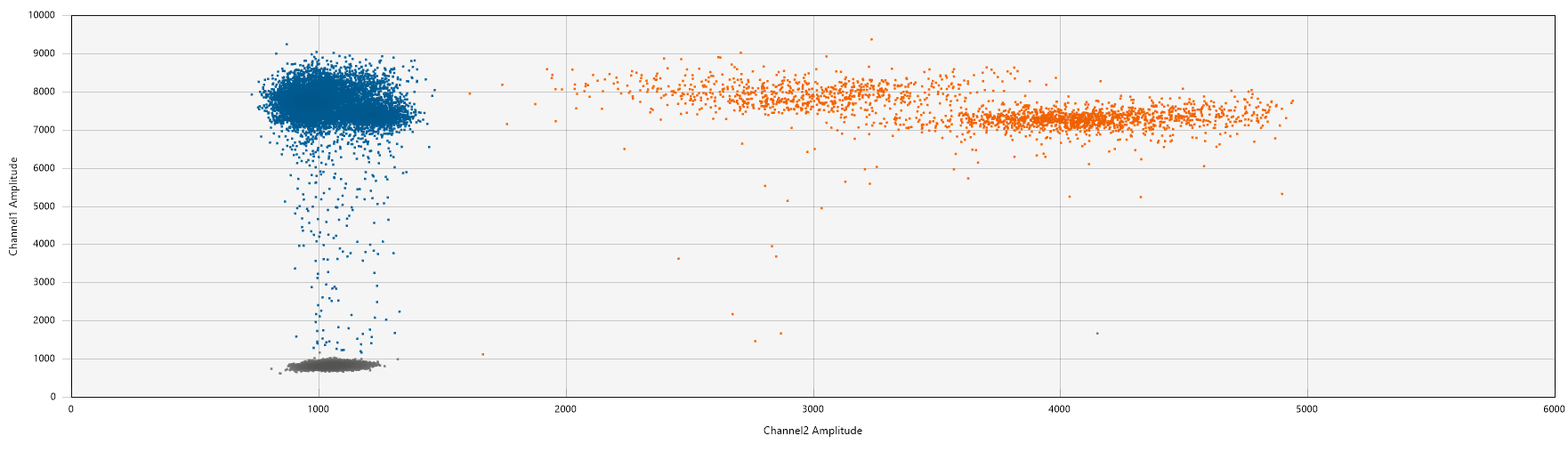

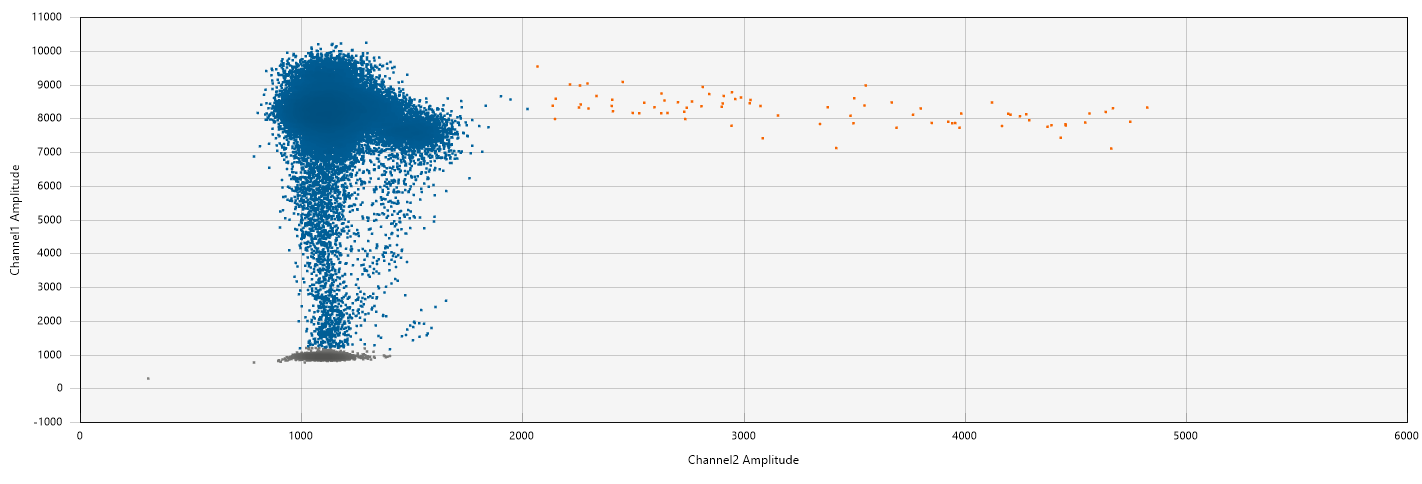


S2- 9:1

S3- 8:2

S1- 10:0

S4- 7:3

S5- 6:4

S6- 5:5

S7- 4:6

S8- 3:7

S9- 2:8

S10- 1:9

S11- 0:10


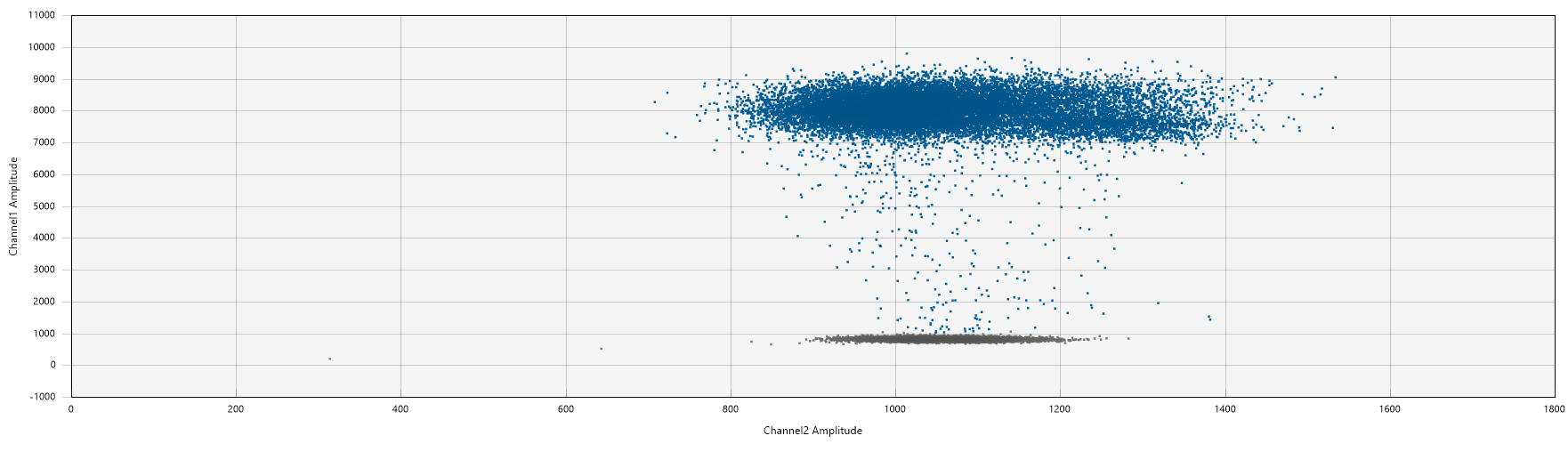

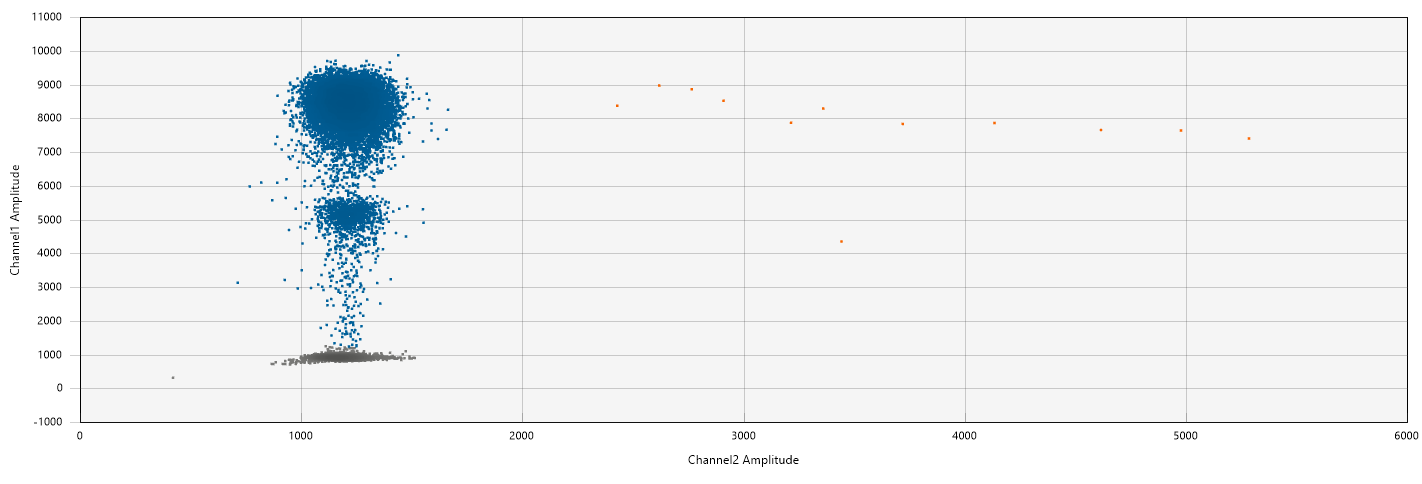

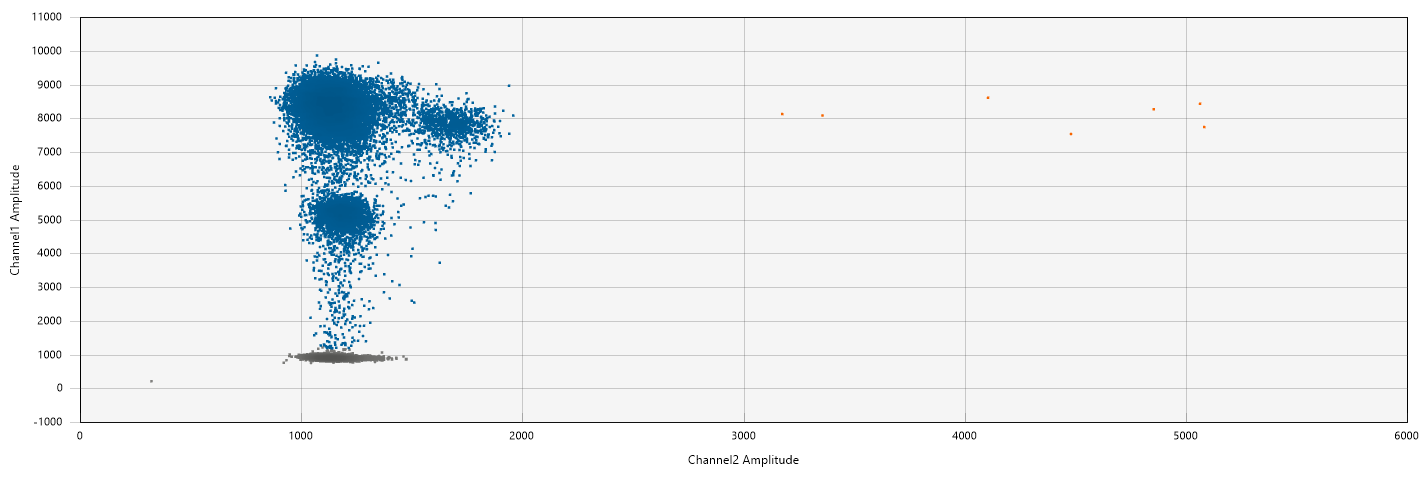

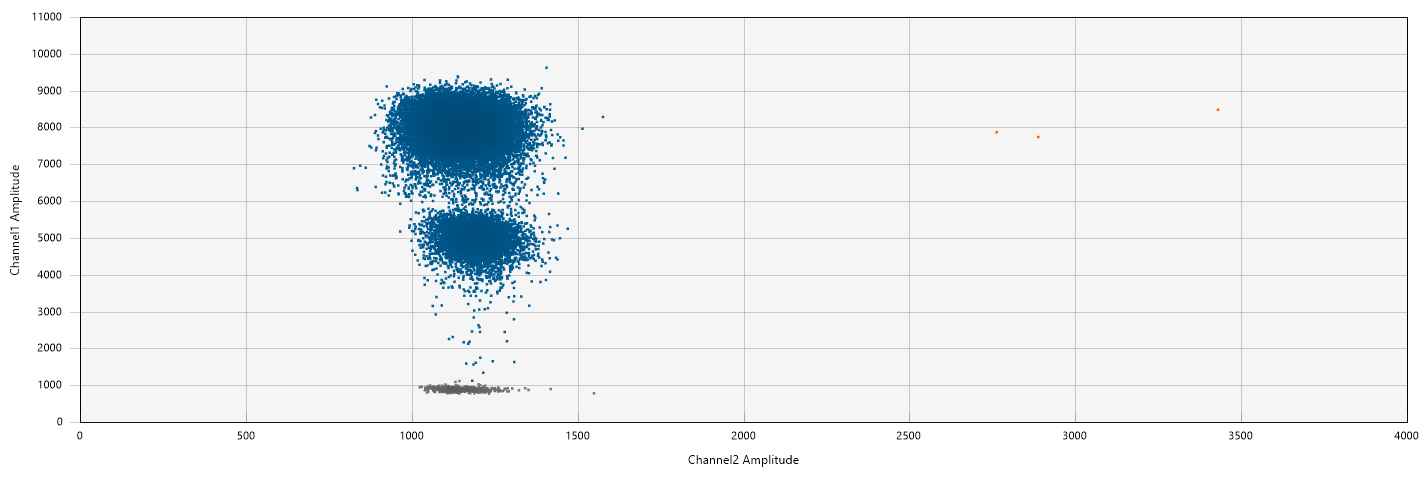

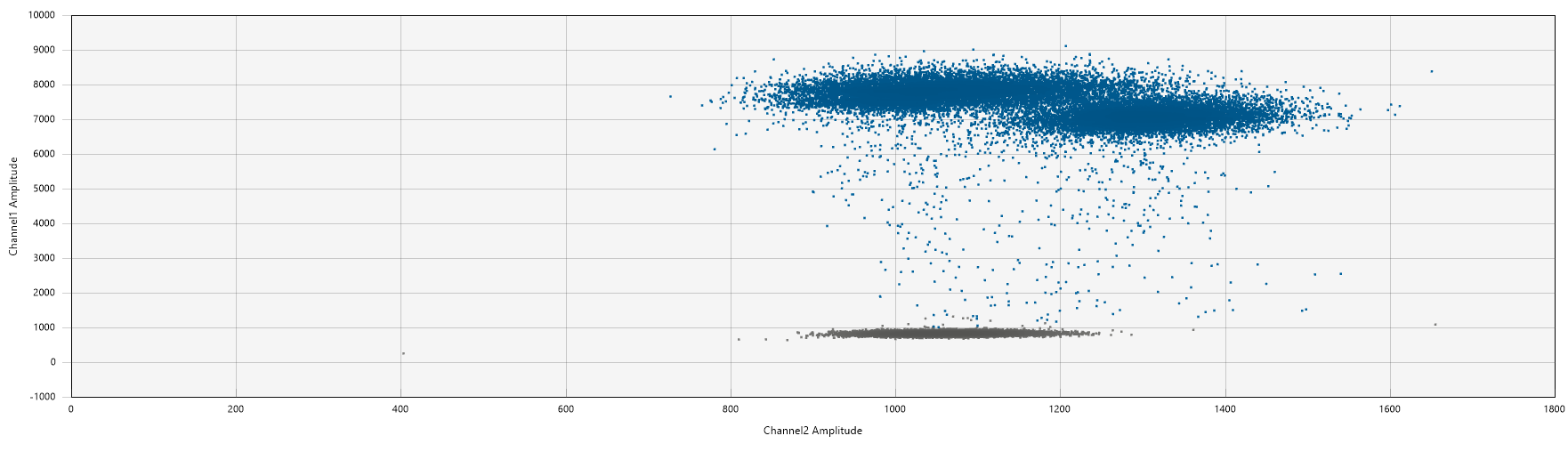

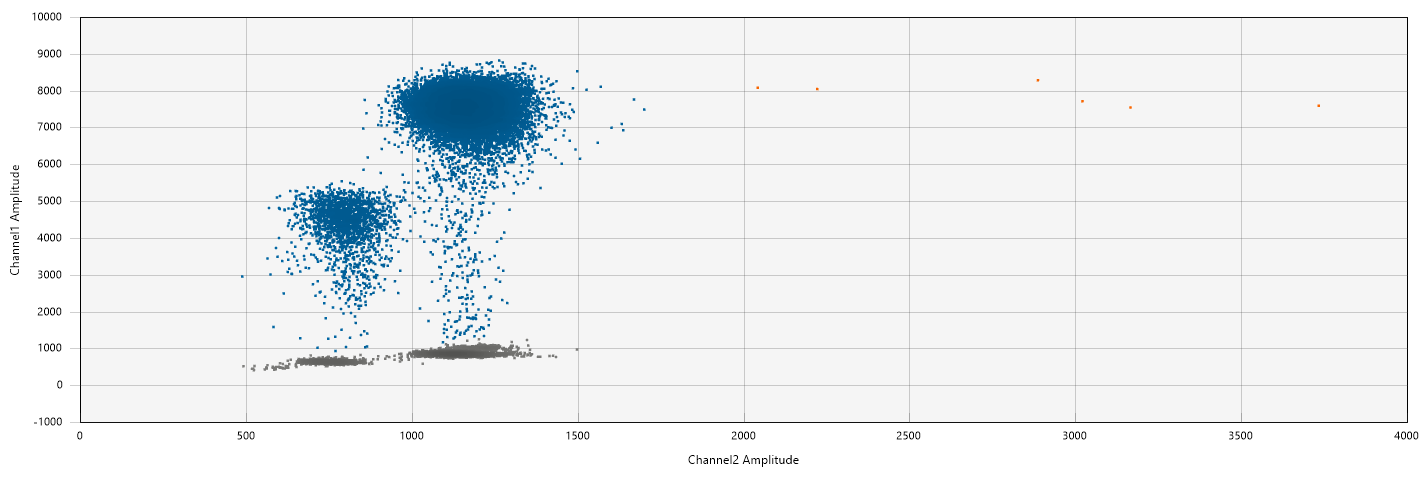

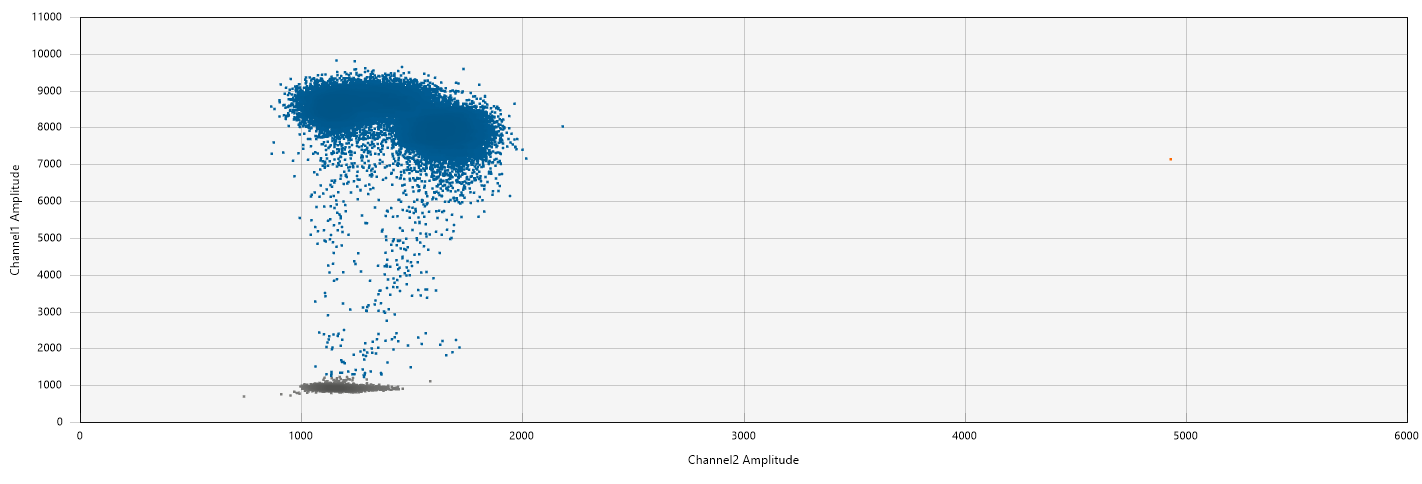

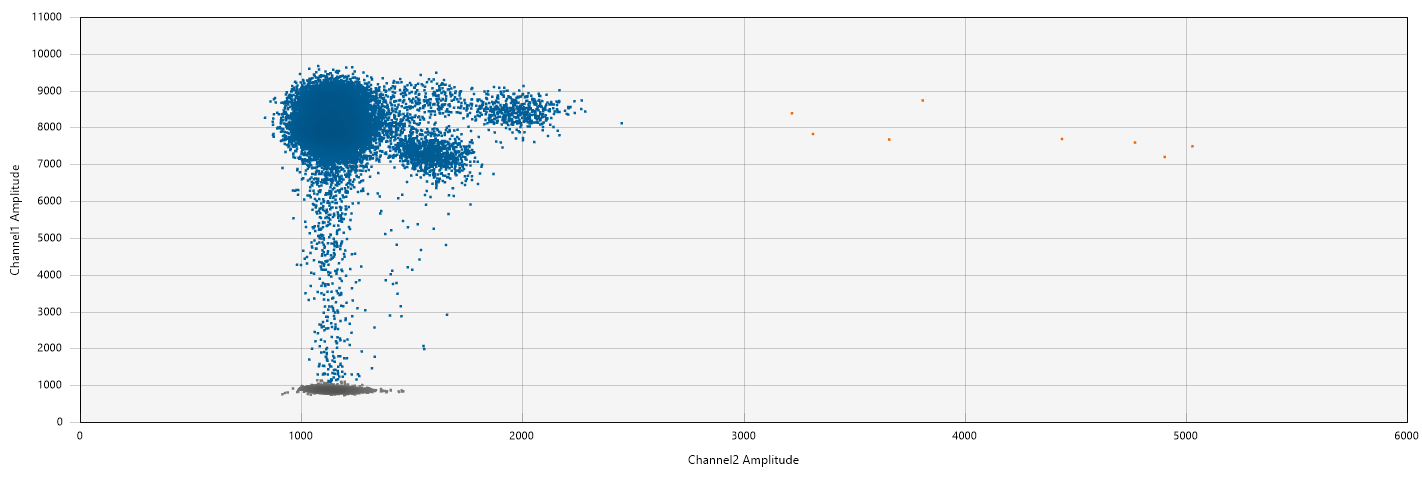

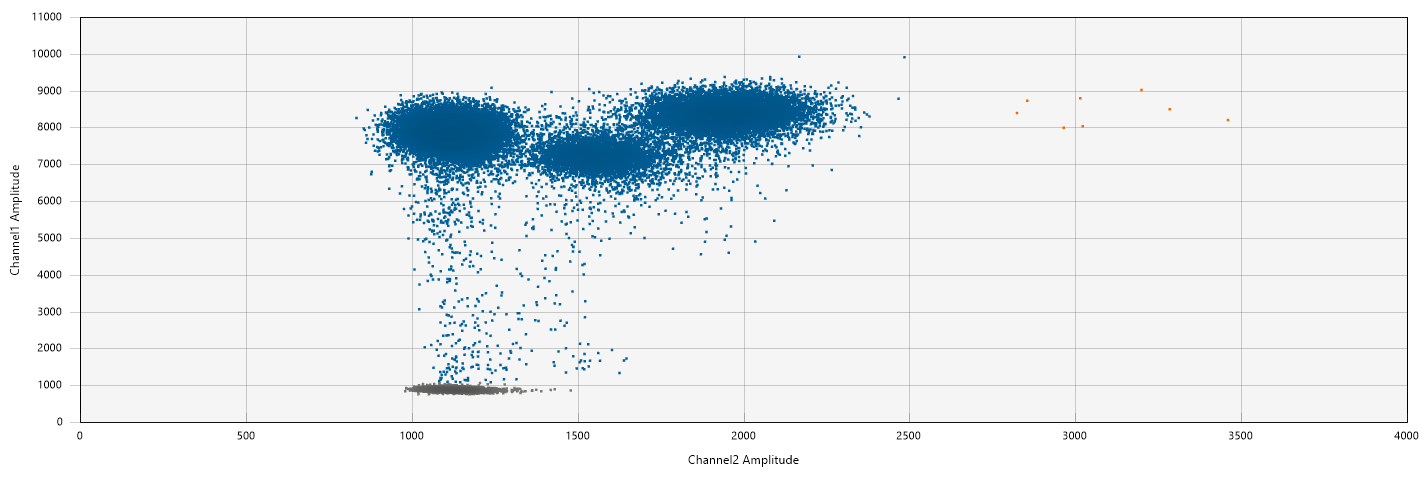

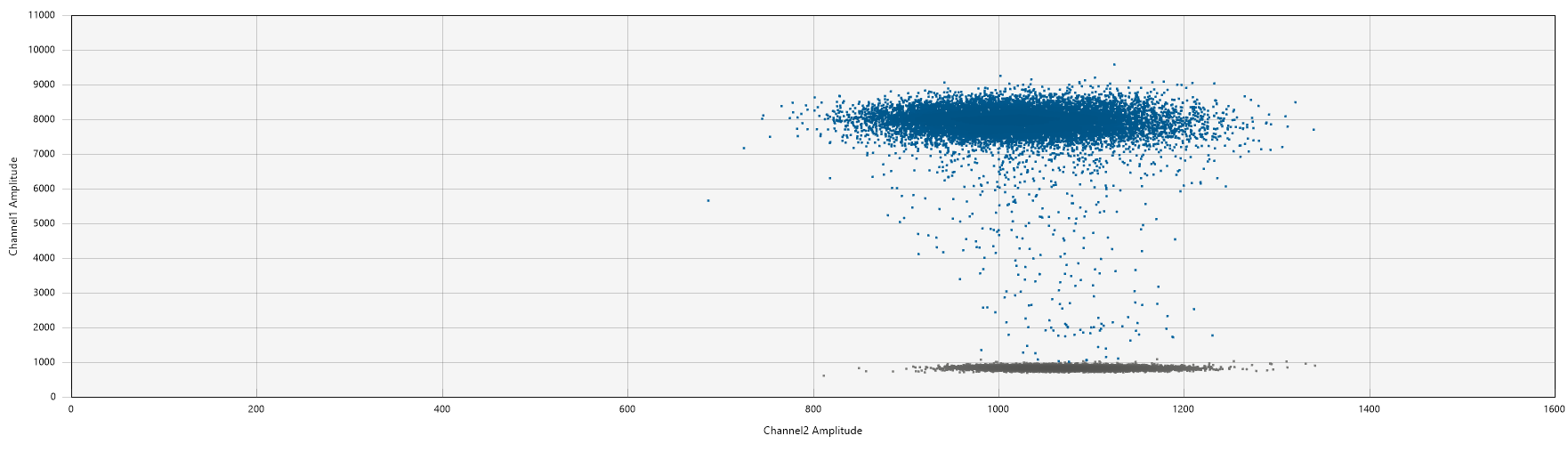

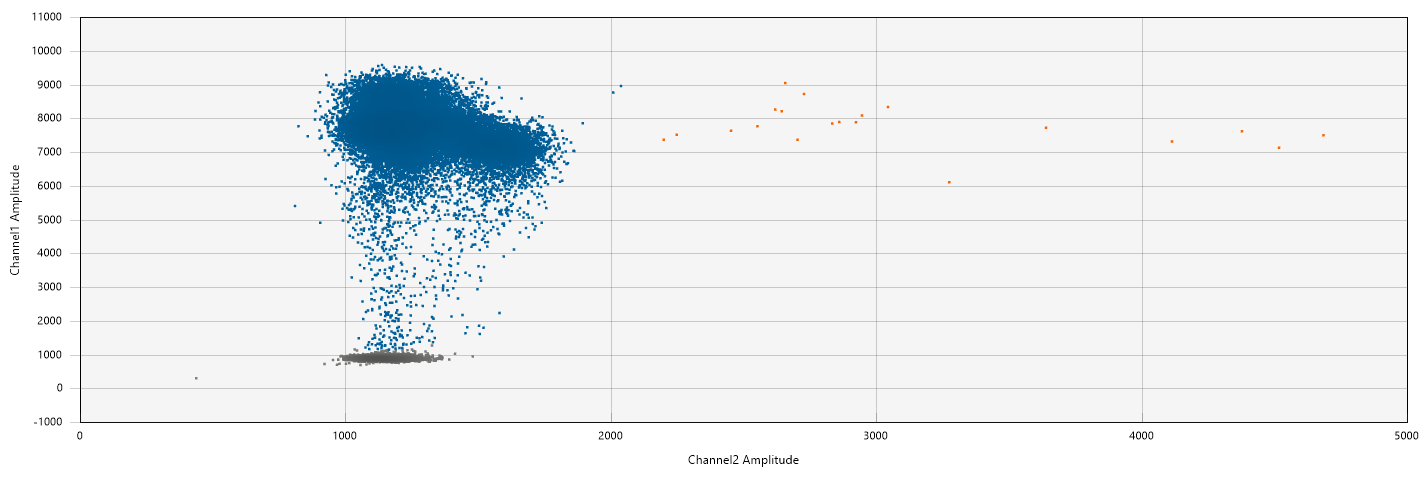

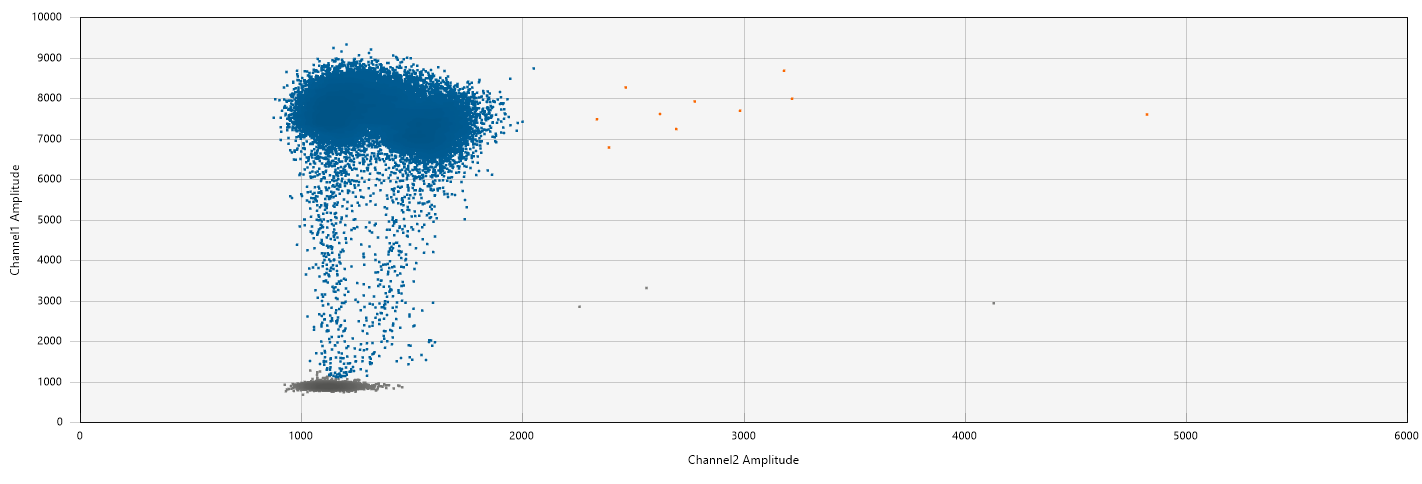

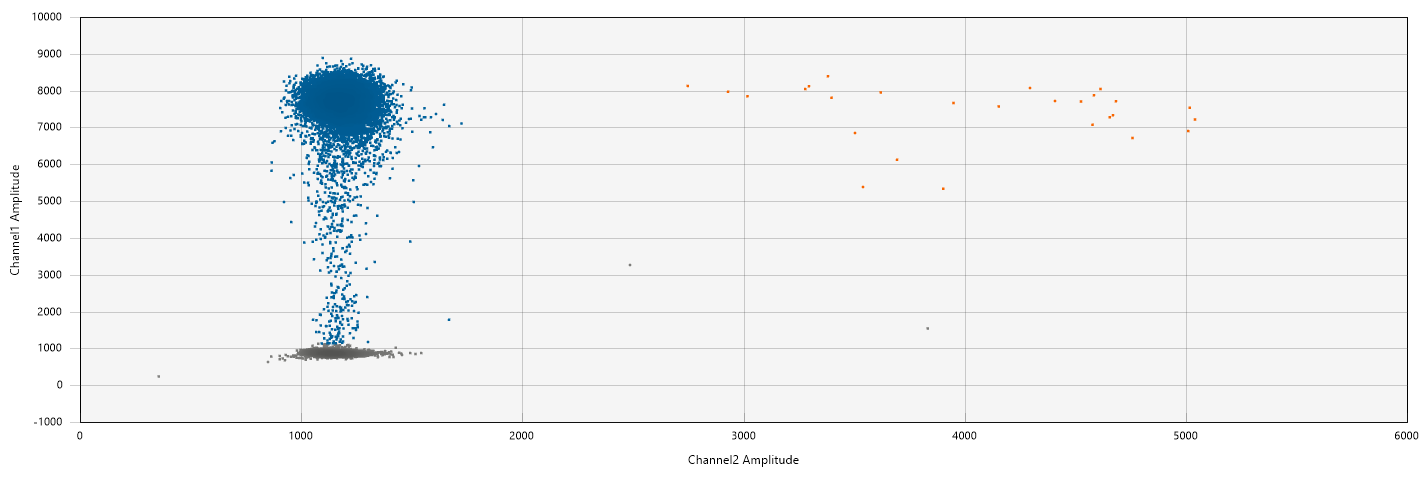

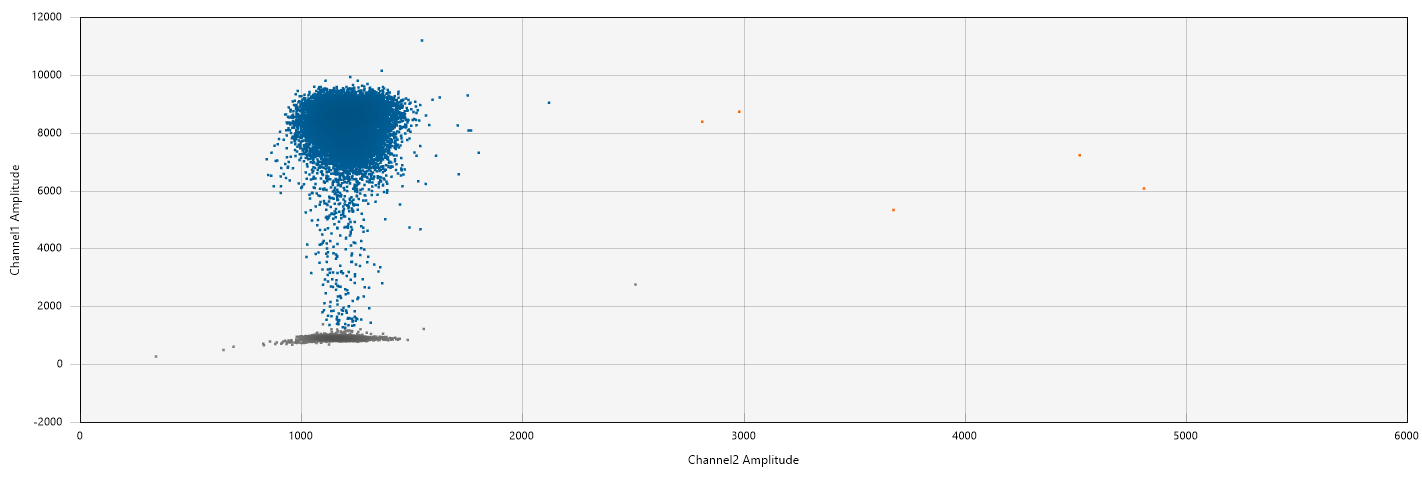

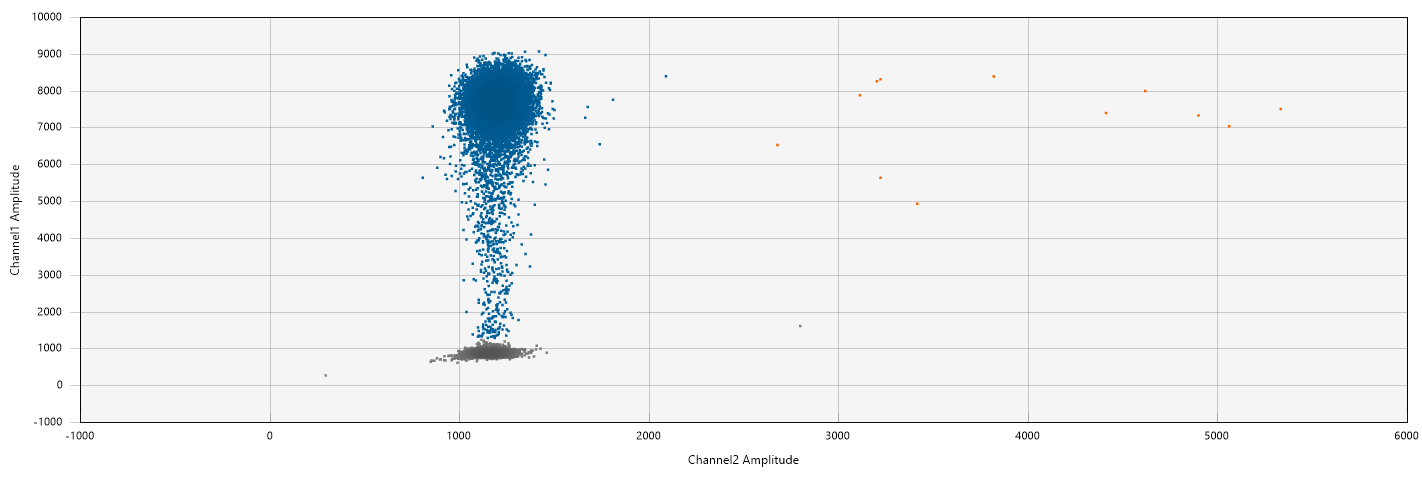


S12: Indel-1

S13: Indel-2

S14: Indel-3

S15: Indel-4

S16: Indel-5

S17: Indel-6

S18: Indel-7

S19: Indel-8

S20: Indel-9

S21: Indel-10

S22: Indel-11

S23: Indel-12

S24: Indel-13

S25: Indel-14

S26: Indel-15
